# Sex-Related Difference in the Association Between Child Neglect and the Accuracy of Body Weight Perception Among Chinese Primary Schoolchildren

**DOI:** 10.3389/fpubh.2021.769604

**Published:** 2021-11-23

**Authors:** Hong-jie Yu, Xiangxiang Liu, Ming-wei Liu, Min-zhe Zhang, Miaobing Zheng, Qi-qiang He

**Affiliations:** ^1^School of Health Sciences, Wuhan University, Wuhan, China; ^2^Shenzhen Second People's Hospital, Shenzhen, China; ^3^The Julius Center for Health Sciences and Primary Care, University Medical Center Utrecht, Utrecht, Netherlands; ^4^Institute for Physical Activity and Nutrition, School of Exercise and Nutrition Sciences, Deakin University, Geelong, VC, Australia; ^5^Hubei Biomass-Resource Chemistry and Environmental Biotechnology Key Laboratory, Wuhan University, Wuhan, China

**Keywords:** child neglect, body weight perception, sex differences, children, multinomial logistic regression

## Abstract

**Introduction:** Body weight perception (BWP) directly determines weight management behaviors. Although child neglect is a well-established risk factor for managing body weight, little is known about its association with the accuracy of BWP. This study aimed to assess the cross-sectional and longitudinal associations between child neglect and BWP accuracy in primary schoolchildren, and explore how these differ based on the sex of the child.

**Methods:** The sample included 1,063 primary schoolchildren (557 boys and 506 girls, aged 8–10 years) from a two-wave observational study between 2018 and 2019 in Wuhan, China. Child neglect and BWP were investigated using self-administered questionnaires. The accuracy of BWP was defined by comparing the BWP and actual body weight, and it was categorized into three groups—consistent, underestimated, and overestimated. Multinomial logistic regression analysis was conducted with fitting child neglect as the independent variable and the accuracy of BWP as the dependent variable.

**Results:** The prevalence of weight misperception was ~44% at baseline (underestimation 40%; overestimation 4%) in Chinese primary schoolchildren. In the cross-sectional analysis, children with a higher level of neglect were more likely to misperceive their body weight. Moreover, there was an apparent sex-related difference that boys who experienced a higher level of neglect significantly reported more underestimation, while girls reported more overestimation. There was no significant longitudinal association between child neglect and the change in BWP accuracy.

**Conclusions:** This study revealed that a higher level of neglect was significantly associated with underestimated BWP in boys and overestimated BWP in girls. The mechanisms of sex-related difference and whether child neglect is involved in the change in BWP, merit further investigations.

## Introduction

Body weight perception (BWP) is conceptualized as how people understand and perceive their body weight (1). It plays an important role in the management of body weight with consciousness and behaviors (2). Studies have revealed that BWP directly influences weight-control behaviors, including physical activities, and dietary behaviors (2–6). On the one hand, the misperception of body weight in those who are underweight, overweight, or obese may reduce the interest in or attempts at weight-control behaviors (5, 7). On the other hand, in those with normal weight, the misperception of body weight fuels the transition to unhealthy lifestyles (1). Given that the prevalence of childhood overweight and obesity is persistently high (over 20%) in China (8), improving the accuracy of BWP may be an effective strategy to control abnormal weight in children.

Body weight perception interacts with the actual body weight, and is affected by a range of social factors (9). Of these, child maltreatment is of particular interest, since it comprehensively reflects the child's growth environment (10). Several systematic reviews have indicated that childhood maltreatment contributed to a lifelong risk for obesity (11–13). However, little is known about the association between child maltreatment and BWP. To the best of our knowledge, only two studies assessed the relationship between childhood maltreatment and the discrepancy of BWP, but both in adults (14, 15). Late childhood to early adolescence is a critical window for physical and psychological development; children and adolescents begin to notice their own body shape and how peers perceive them (16). Therefore, an investigation into the association between child maltreatment and BWP during this period is warranted.

Additionally, sex was closely related to BWP, where underestimation of body weight is more common in boys, while overestimation is more common in girls (17, 18). Furthermore, there were differences in child maltreatment, in that boys were more likely to experience higher levels of emotional and physical neglect than girls (19). These findings suggested that the association between child maltreatment and BWP might differ depending on sex. Child maltreatment is a multidimensional concept that includes physical and sexual abuse, and neglect. Of these, child neglect is the most prevalent subtype, but received the least concern in the current study (20, 21). Given the high burden of child neglect (the prevalence was nearly 50%) (21) and inaccurate BWP (the prevalence of misperception was over 30%) (17, 22), we conducted a two-wave observational study to examine the cross-sectional and longitudinal associations between child neglect and the accuracy of BWP, and assess whether those vary by sex.

## Methods

### Study Design

This study was originally designed to investigate the health outcomes of child neglect in a convenience sample in four public primary schools in Wuhan, China. All students from grades 3 to 4 were invited to participate in the baseline study in April 2018, and would be followed-up with annually until graduation from primary school (grade 6). Written informed consent was obtained from children and parents. This study was approved by the Medical Research Ethics Committee of Wuhan University.

### Participants

A total of 1,340 children (aged 9.1 ± 0.6 years) were included at baseline following the inclusion criteria: (i) without preexisting congenital diseases and defects (e.g., diabetes, heart disease, hypertension, and disability); (ii) not being transfer students or the transfer period was over one semester; and (iii) did not suffer major family changes (e.g., parents' divorce and relative's death). This study used the data of baseline and the first wave of follow-up (2019) for data analysis. A total of 1,193 children completed the two-wave observation, and 147 children were lost to follow-up (attrition rate: 11%) because of the following reasons: sick/compassionate leave, school transfer, and refusal to partake in the follow-up investigation.

### Measurements

Body weight: Children's height without shoes and with light clothes was measured using standard methods to calculate BMI, with weight (*kg*) divided by height squared (*m*^2^) (23). The actual body weight was defined as severe underweight, underweight, normal weight, overweight, and obese, using the BMI reference values for Chinese school-age children (24).

Body weight perception: BWP was determined by asking “Which body shape do you think you have?” There were five possible responses: “too thin,” “relatively thin,” “all right,” “relatively heavy,” and “too heavy” (16). The accuracy of BWP was examined by comparing the actual body weight with the BWP. Children who were (severely) underweight but perceived themselves as “all right” and “relatively (too) heavy” and those with normal weight who perceived themselves as “relatively (too) heavy” were categorized as “overestimated.” Conversely, children who were overweight or obese but perceived themselves as “all right” and “too (relatively) thin” and those with normal weight who perceived themselves as “relatively (too) thin” were categorized as “underestimated.” Children who are (severely) underweight and perceived themselves as “relatively thin” or “too thin,” who were normal weight and perceived themselves as “all right,” and those who were overweight or obese and perceived themselves as “relatively heavy” or “too heavy,” were categorized under “consistent” (25).

Child neglect: Child neglect was assessed using the Child Neglect Scale (CNS) developed by Yang et al. (26), which comprises 38 items in four dimensions: physical neglect (7 items); emotional neglect (14 items); security neglect (9 items); and communication neglect (8 items). Each item was evaluated using a four-point Likert scale: “never,” “occasional,” “usual,” and “constant.” The CNS has been validated in Chinese schoolchildren and has shown good internal consistency (Cronbach's α = 0.85) and test-retest reliability (0.90) (27). The total score of the CNS ranges from 4 to 152, with a higher score indicating a higher level of neglect. This study used the tertiles of neglect for analysis.

Covariates: Age and sex were reported by children. Demographic characteristics, including monthly household income, parental education, and single-child family were reported by parents. Monthly household income was classified as “ <10,000,” “10,000–20,000,” and “>20,000” (*RMB*). Parental education was classified as “middle school or lower,” “high school,” and “university or higher.” The single-child family was determined with a “yes” and “no.” The pubertal stage was evaluated in a private room by trained investigators using the Tanner Stage Scale. The pubertal development was determined by comparing the external primary and secondary sex characteristics of children with the realistic color image of the Tanner Stage Scale (28).

### Procedures

Body weight perception and child neglect were assessed in classrooms with the guidance of teachers, using self-administered questionnaires. All questionnaires were answered independently to protect privacy and were signed with real names for tracking during the follow-up. Trained investigators read and explained each item in the questionnaires to facilitate completion by the children. The children were then invited to attend the anthropometric test and pubertal stage evaluation in separate locations, according to sex. The demographic information was also collected using questionnaires, which were brought home by children.

### Statistical Analysis

Among the 1,193 children who completed the baseline and follow-up surveys, 130 were excluded from the final analysis because of missing information in child neglect, actual body weight, BWP, or other covariates. Chi-square test was used to examine the difference in all variables between sex and between the included and excluded samples. All descriptive statistics of 1,063 children (557 boys and 506 girls) were categorical variables and presented as frequency (%). Multinomial logistical regression model regarding the consistent BWP as reference group was used to examine the cross-sectional and longitudinal association between child neglect, and the accuracy of BWP stratified by sex. For cross-sectional analyses, model 1 was adjusted for school, age, monthly household income, parental education, and single-child family at baseline, and model 2 was further adjusted for pubertal stage and BMI at baseline. For longitudinal analyses, BWP at baseline was further included in model 1. The threshold for the statistical significance was set at *P*-value < 0.05. Statistical Package for the Social Sciences (SPSS) software (version 20.0. Armonk, NY: IBM Corp.) was used to conduct all analyses.

## Results

As shown in Table 1, the children's age ranged from 8 to 10 years. Approximately 70% of the children lived in a household with a monthly income of over 10,000 yuan, or parents with a university degree or higher. More than half of the children had no siblings in the family. There were no significant differences in demographic characteristics between sex. Boys had a higher level of child neglect than girls (tertiles 2–3: 66.4 vs. 59.1%). The prevalence of underweight and overweight children was ~3 and 20%, respectively, in all children at baseline, while the prevalence of overweight boys (23%) was much higher than overweight girls (15.6%). The prevalence of underweight and overweight children was 1.4 and 25.6%, respectively, in all children during follow-up, and the sex-related difference was still significant. Nearly half of the children perceived their body weight as too (relatively) thin/heavy at baseline and follow-up, with no significant sex-related difference. Approximately 40% of the children did not perceive their body weight accurately at baseline and follow-up. Among them, underestimation was more common than overestimation, regardless of sex. Sex-related difference in BWP accuracy was insignificant at baseline, but significant at follow-up. There were no significant differences in all variables between the included and excluded samples, except for a marginally significant lower level of neglect among children in the excluded sample (Supplementary Table 1).

**Table 1:** Descriptive characteristics in the sample of 1,063 primary schoolchildren.

**Characteristics**	**Total (*n* = 1,063)**	**Boys (*n* = 557)**	**Girls (*n* = 506)**	***P* ^**a**^**
**Baseline**				
**Age**, ***years***				0.223
8	114 (10.7%)	55 (9.9%)	59 (11.7%)	
9	725 (68.2%)	374 (67.1%)	351 (69.4%)	
10	224 (21.1%)	128 (23%)	96 (19%)	
**Monthly household income**, ***yuan***				0.326
<10,000	326 (30.7%)	176 (31.6%)	150 (29.6%)	
10,000~20,000	457 (43%)	245 (44%)	212 (41.9%)	
>20,000	280 (26.3%)	136 (24.4%)	144 (28.5%)	
**Parental education**				0.952
Middle school or lower	95 (8.9%)	49 (8.8%)	46 (9.1%)	
High school	163 (15.3%)	84 (15.1%)	79 (15.6%)	
University or higher	805 (75.7%)	424 (76.1%)	381 (75.3%)	
**Single-child family**, ***yes***	575 (54.1%)	312 (56.0%)	263 (52.0%)	0.187
**Pubertal stage**				0.554
Tanner 1	46 (4.3%)	26 (4.7%)	20 (4.0%)	
Tanner 2	565 (53.2%)	302 (54.2%)	263 (52%)	
Tanner 3	451 (42.4%)	228 (40.9%)	223 (44.1%)	
Tanner 4^b^	1 (0.1%)	1 (0.2%)	–	
**Child neglect, tertiles**				0.046
T1 (~54)	394 (37.1%)	187 (33.6%)	207 (40.9%)	
T2 (54–69)	327 (30.8%)	182 (32.7%)	145 (28.7%)	
T3 (70~)	342 (32.2%)	188 (33.8%)	154 (30.4%)	
**Actual body weight**				0.009
Underweight	32 (3%)	15 (2.7%)	17 (3.4%)	
Normal weight	824 (77.5%)	414 (74.3%)	410 (81.0%)	
Overweight	207 (19.5%)	128 (23.0%)	79 (15.6%)	
**Self-perceived body weight**				0.221
Too (relatively) thin	368 (34.6%)	194 (34.8%)	174 (34.4%)	
Normal	550 (51.7%)	278 (49.9%)	272 (53.8%)	
Too (relatively) heavy	145 (13.6%)	85 (15.3%)	60 (11.9%)	
**The accuracy of body weight perception**				0.090
Consistent	592 (55.7%)	312 (56%)	280 (55.3%)	
Underestimated	427 (40.2%)	229 (41.1%)	198 (39.1%)	
Overestimated	44 (4.1%)	16 (2.9%)	28 (5.5%)	
**Follow-up**				
**Actual body weight**				0.010
Underweight	15 (1.4%)	8 (1.4%)	7 (1.4%)	
Normal weight	776 (73%)	385 (69.1%)	391 (77.3%)	
Overweight	272 (25.6%)	164 (29.4%)	108 (21.3%)	
**Self-perceived body weight**				0.456
Too (relatively) thin	271 (25.5%)	150 (26.9%)	121 (23.9%)	
Normal	598 (56.3%)	304 (54.6%)	294 (58.1%)	
Too (relatively) heavy	194 (18.3%)	103 (18.5%)	91 (18%)	
**The accuracy of body weight perception**				0.007
Consistent	641 (60.3%)	327 (58.7%)	314 (62.1%)	
Underestimated	373 (35.1%)	213 (38.2%)	160 (31.6%)	
Overestimated	49 (4.6%)	17 (3.1%)	32 (6.3%)	

a *The sex difference was tested by χ^2^ test*.

b *Tanner 4 was not included in the χ^2^ test*.

Table 2 shows the cross-sectional analysis between child neglect and the accuracy of BWP in all children or stratified by sex. In the overall sample, children in the highest tertile of neglect were more likely to report underestimation (OR = 1.59, 95% CI: 1.17–2.17, *P* = 0.003) and overestimation (OR = 2.19, 95% CI: 1.01–4.74, *P* = 0.047) of their own body weight compared with those in the first tertile, even when adjusted for demographical characteristics, pubertal stage, and BMI. However, the sex-stratified analysis indicated that boys in the highest tertile of neglect showed increased odds of underestimating their body weight (OR = 1.80, 95% CI: 1.16–2.80, *P* = 0.009), while the association between child neglect and underestimated BWP was not significant in girls (OR = 1.35, 95% CI: 0.86–2.12, *P* = 0.189). Conversely, there was a significant association between child neglect and overestimated BWP in girls (OR = 3.36, 95% CI: 1.18–9.57, *P* = 0.023) for the highest tertile of child neglect, which was not significant in boys (OR = 1.01, 95% CI: 0.30–3.38, *P* = 0.991).

**Table 2:** Cross-sectional analysis for the association of child neglect with the accuracy of body weight perception at baseline.

**Child neglect, tertiles**	**Underestimated vs. consistent**						**Overestimated vs. consistent**					
	**Total (*****n*** **=** **1,063)**		**Boys (*****n*** **=** **557)**		**Girls (*****n*** **=** **506)**		**Total (*****n*** **=** **1,063)**		**Boys (*****n*** **=** **557)**		**Girls (*****n*** **=** **506)**	
	**OR (95% CI)**	***P***	**OR (95% CI)**	***P***	**OR (95% CI)**	***P***	**OR (95% CI)**	***P***	**OR (95% CI)**	***P***	**OR (95% CI)**	***P***
**Model 1** ^a^												
T1 (~54)	1.00		1.00		1.00		1.00		1.00		1.00	
T2 (54–69)	1.11 (0.81–1.51)	0.515	1.21 (0.78–1.88)	0.389	0.99 (0.63–1.56)	0.970	1.51 (0.68–3.38)	0.312	0.61 (0.16–2.33)	0.473	2.41 (0.83–6.99)	0.104
T3 (70~)	1.63 (1.20–2.22)	**0.002**	1.79 (1.16–2.77)	**0.009**	1.41 (0.91–2.21)	0.127	2.25 (1.04–4.85)	**0.040**	0.99 (0.30–3.32)	0.992	3.51 (1.24–9.96)	**0.018**
**Model 2** ^b^												
T1 (~54)	1.00		1.00		1.00		1.00		1.00		1.00	
T2 (54–69)	1.11 (0.82–1.52)	0.500	1.23 (0.79–1.91)	0.355	1.00 (0.63–1.58)	0.990	1.53 (0.68–3.41)	0.304	0.60 (0.16–2.29)	0.453	2.44 (0.84–7.08)	0.101
T3 (70~)	1.59 (1.17–2.17)	**0.003**	1.80 (1.16–2.80)	**0.009**	1.35 (0.86–2.12)	0.189	2.19 (1.01–4.74)	**0.047**	1.01 (0.30–3.38)	0.991	3.36 (1.18–9.57)	**0.023**

a *Model 1 adjusted for school, age, sex (sex stratified analysis did not include sex), monthly household income, parental education, and single-child family at baseline*.

b *Model 2 further adjusted for pubertal stage and body mass index at baseline. Bold values indicate statistical significance with P-value < 0.05*.

The accuracy of BWP at follow-up, with child neglect at baseline, was also examined (Table 3). However, child neglect at baseline was not associated with the misperception of body weight at follow-up, in both overall and sex-stratified analyses.

**Table 3:** Longitudinal analysis for the association of child neglect with the accuracy of body weight perception at follow-up.

**Child neglect, tertiles**	**Underestimated vs. consistent**						**Overestimated vs. consistent**					
	**Total (*****n*** **=** **1,063)**		**Boys (*****n*** **=** **557)**		**Girls (*****n*** **=** **506)**		**Total (*****n*** **=** **1,063)**		**Boys (*****n*** **=** **557)**		**Girls (*****n*** **=** **506)**	
	**OR (95% CI)**	***P***	**OR (95% CI)**	***P***	**OR (95% CI)**	***P***	**OR (95% CI)**	***P***	**OR (95% CI)**	***P***	**OR (95% CI)**	***P***
**Model 1** ^a^												
T1 (~54)	1.00		1.00		1.00		1.00		1.00		1.00	
T2 (54–69)	1.09 (0.79–1.53)	0.596	1.50 (0.95–2.38)	0.085	0.75 (0.45–1.23)	0.250	1.12 (0.52–2.41)	0.783	0.81 (0.23–2.87)	0.740	1.16 (0.43–3.12)	0.765
T3 (70~)	0.94 (0.67–1.31)	0.697	1.18 (0.74–1.88)	0.493	0.73 (0.45–1.19)	0.204	1.49 (0.73–3.07)	0.277	0.85 (0.25–2.94)	0.800	1.77 (0.70–4.44)	0.228
**Model 2** ^b^												
T1 (~54)	1.00		1.00		1.00		1.00		1.00		1.00	
T2 (54–69)	1.10 (0.78–1.53)	0.593	1.55 (0.97–2.47)	0.065	0.72 (0.43–1.18)	0.192	1.13 (0.52–2.44)	0.761	0.89 (0.25–3.21)	0.856	1.15 (0.43–3.09)	0.782
T3 (70~)	0.90 (0.64–1.26)	0.524	1.15 (0.71–1.84)	0.578	0.68 (0.41–1.12)	0.131	1.46 (0.71–3.02)	0.306	0.87 (0.25–3.01)	0.822	1.75 (0.69–4.45)	0.239

a *Model 1 adjusted for school, age, sex (sex stratified analysis did not include sex), monthly household income, parental education, single-child family, and the accuracy of body weight perception at baseline*.

b *Model 2 further adjusted for pubertal stage and body mass index at baseline*.

## Discussions

There was a high discrepancy between BWP and actual body weight in Chinese schoolchildren, with nearly 40% of the children reporting underestimation, and 5% reporting overestimation. This study revealed a significant cross-sectional association between child neglect and underestimated and overestimated BWP. Moreover, the association differed in sex that boys with higher neglect were more likely to report underestimated BWP, while girls with higher neglect were more likely to report overestimated BWP. However, longitudinal associations were not significant in both overall and sex-stratified analyses.

The prevalence of inaccurate BWP was nearly 45% in our study, which was slightly higher than that in previous studies among Chinese schoolchildren (17, 18, 29). Comparing those studies, the prevalence of the underestimated BWP was consistently much higher than that of the overestimated BWP (17, 18, 29), and the magnitude varied from ~2 times higher (25.3% underestimated BWP vs. 13.1% overestimated BWP) in the study of Wang VH et al. (18) to 10 times higher than (about 40% underestimated BWP vs. 4% overestimated BWP) in our study. This was mainly due to the difference in the study setting that all children in our study lived in urban areas, while 63% of the children in a study by Wang Y et al. (17) and 47% of the children in a study by Wang (18) lived in rural areas. Therefore, rural-urban variation may affect the difference between the prevalence of the underestimated and overestimated BWP. Additionally, a similar significant sex-related difference was observed in a study by Wang Y et al. (17), while Wang VH et al. (18) reported an insignificant sex-related difference in children aged 6–11 years. They consistently explained that the sex-related difference may result from different beliefs of ideal body image, in that boys preferred masculinity and girls preferred femininity (17, 18). Furthermore, Wang infers that the prevalence of the underestimated and overestimated BWP may change a lot from childhood to adolescence in girls, and that a higher proportion of girls will overestimate their body weight (18). Therefore, future weight management programs for children and adolescence should consider the sex-related difference in the different growth and developmental stages.

Currently, no study examines the association between child neglect and inaccurate BWP in children, while two studies in adults consistently indicated that child maltreatment was associated with inaccurate BWP in adulthood (14, 15). These studies suggested that adults with higher child maltreatment may be more sensitive and vulnerable to subsequent stressors and consequently, easily misperceive their body weight (14, 15). In addition, as for children, the maltreatment experience directly leads to maladjustment, including depression and low self-esteem (30). In turn, the adverse psychological consequence was associated with inaccurate BWP (16, 31). Additionally, child neglect in families inherently results from parents/caregivers. The lack of supervision and communication between children and parents may lead to children's BWP is more likely influenced by peers and social media (17). On the other hand, the lack of attachment and security caused by neglect exacerbates the parent-child discrepancy in children's BWP. A study by Uccula et al. indicated that parents of children with insecurities had a tendency to underestimate the actual body weight of their children, particularly those who were overweight or obese (32). In this situation, parents believe that their children perceive themselves as more obese than the parents perceive them, and unconsciously construct an obesogenic family environment (32).

Importantly, our study highlighted the sex-related difference in the association between child neglect and misperception of body weight, which was also consistent with the findings of two studies in adults (14, 15). These studies consistently explained that females with a higher level of child maltreatment were more vulnerable to life stress (14, 15), and it might be fueled by maladaptive strategies that used body shape to determine self-worth (14). However, it was unclear whether the explanation could be applied to children. In addition, children's coping strategies with stress differ in sex, in that girls were more likely to suffer from depression after relational victimization (33). Moreover, a study by Wei et al. indicated that girls who experienced childhood trauma displayed a higher risk of depression than boys (34). Additionally, we failed to identify a longitudinal significant association between child neglect and the accuracy of BWP. We supposed that the measurement of body weight might have an influence on children's BWP and that the preface of the parents' questionnaire reminded them to focus on children's body weight.

### Strengths and Limitations

To the best of our knowledge, our study was the first to investigate the association between child neglect and the accuracy of BWP using a longitudinal design. Our findings directly indicated the negative influence of child neglect on the accuracy of BWP, which indirectly emphasized that the development of BWP and the management of body weight required family support. Additionally, the identification of sex-related differences in their association will be helpful for explaining the sex-related difference in the prevalence of childhood obesity and the inaccuracy of BWP in children in China.

This study has several limitations. First, this study used a convenience sampling method, with a high proportion of children from high socioeconomic families. Second, four public schools were in the urban areas. Therefore, the generalizability of our results is limited by sample selection bias. Third, ~11% of the children were excluded from the final analysis, which might also result in selection bias. However, the comparison of all variables between the included and excluded samples was insignificant. Fourth, child neglect was assessed using a self-reported questionnaire, making a recall bias inevitable. However, CNS has been validated and has shown good reliability in Chinese schoolchildren. Finally, other potential confounders to BWP, including exposure to social media and psychological factors, were not assessed in the analysis.

## Conclusions

This study suggested that the prevalence of inaccurate BWP was common in Chinese primary schoolchildren, and largely consisted of underestimated BWP. Importantly, our study revealed that child neglect was significantly associated with inaccurate BWP. Moreover, a significant sex-related difference was identified that boys who experienced a higher level of neglect were more likely to report underestimated BWP, while girls who had been neglected were more likely to report overestimated BWP. The mechanisms of sex-related difference and whether child neglect is involved in the change in BWP merit further investigations.

## Data Availability Statement

The raw data supporting the conclusions of this article will be made available by the authors, without undue reservation.

## Ethics Statement

The studies involving human participants were reviewed and approved by the Medical Research Ethics Committee of Wuhan University. Written informed consent to participate in this study was provided by the participants' legal guardian/next of kin.

## Author Contributions

H-jY: conceptualization, data curation, formal analysis, investigation, methodology, validation, visualization, writing—original draft, and writing—review and editing. XL: conceptualization, formal analysis, methodology, writing—original draft, and writing—review and editing. M-wL: investigation, project administration, and writing-review and editing. MZ: methodology and writing—review and editing. Q-qH: conceptualization, data curation, funding acquisition, investigation, methodology, project administration, resources, software, supervision, validation, and writing-review and editing. All authors have read and approved the final manuscript.

## Funding

HY was supported by the Special Fund Support for Postgraduate Overseas Exchange Program of Wuhan University and XL was supported by the China Postdoctoral Science Foundation (2021M692215) and the Guangdong Basic and Applied Basic Research Foundation (2020A1515111134).

## Conflict of Interest

The authors declare that the research was conducted in the absence of any commercial or financial relationships that could be construed as a potential conflict of interest.

## Publisher's Note

All claims expressed in this article are solely those of the authors and do not necessarily represent those of their affiliated organizations, or those of the publisher, the editors and the reviewers. Any product that may be evaluated in this article, or claim that may be made by its manufacturer, is not guaranteed or endorsed by the publisher.
